# Blink-Related Oscillations Provide Naturalistic Assessments of Brain Function and Cognitive Workload within Complex Real-World Multitasking Environments

**DOI:** 10.3390/s24041082

**Published:** 2024-02-07

**Authors:** Cleo Page, Careesa Chang Liu, Jed Meltzer, Sujoy Ghosh Hajra

**Affiliations:** 1 Division of Engineering Science, University of Toronto, Toronto, ON M5S 2E4, Canada; 2 Department of Biomedical Engineering and Science, Florida Institute of Technology, 150 W University Boulevard, Melbourne, FL 32901, USA; liuc@fit.edu; 3 Baycrest Health Sciences, Toronto, ON M6A 2E1, Canada

**Keywords:** neuroergonomics, Multi-Attribute Task Battery (MATB), blink, blink-related oscillations, electroencephalography (EEG), pilots, cognitive state, human machine interaction

## Abstract

Background: There is a significant need to monitor human cognitive performance in complex environments, with one example being pilot performance. However, existing assessments largely focus on subjective experiences (e.g., questionnaires) and the evaluation of behavior (e.g., aircraft handling) as surrogates for cognition or utilize brainwave measures which require artificial setups (e.g., simultaneous auditory stimuli) that intrude on the primary tasks. Blink-related oscillations (BROs) are a recently discovered neural phenomenon associated with spontaneous blinking that can be captured without artificial setups and are also modulated by cognitive loading and the external sensory environment—making them ideal for brain function assessment within complex operational settings. Methods: Electroencephalography (EEG) data were recorded from eight adult participants (five F, M = 21.1 years) while they completed the Multi-Attribute Task Battery under three different cognitive loading conditions. BRO responses in time and frequency domains were derived from the EEG data, and comparisons of BRO responses across cognitive loading conditions were undertaken. Simultaneously, assessments of blink behavior were also undertaken. Results: Blink behavior assessments revealed decreasing blink rate with increasing cognitive load (*p* < 0.001). Prototypical BRO responses were successfully captured in all participants (*p* < 0.001). BRO responses reflected differences in task-induced cognitive loading in both time and frequency domains (*p* < 0.05). Additionally, reduced pre-blink theta band desynchronization with increasing cognitive load was also observed (*p* < 0.05). Conclusion: This study confirms the ability of BRO responses to capture cognitive loading effects as well as preparatory pre-blink cognitive processes in anticipation of the upcoming blink during a complex multitasking situation. These successful results suggest that blink-related neural processing could be a potential avenue for cognitive state evaluation in operational settings—both specialized environments such as cockpits, space exploration, military units, etc. and everyday situations such as driving, athletics, human-machine interactions, etc.—where human cognition needs to be seamlessly monitored and optimized.

## 1. Introduction

With the increasing complexity and automation of modern life, there is great interest in better understanding human cognition and designing systems to better accommodate and optimize human performance in complex work environments [1,2,3]. From specialized fields like aviation and surgery to everyday activities such as driving, there is an ever-increasing demand on the brain’s ability to simultaneously process complex sensory inputs such as dynamic auditory, visual, and tactile stimuli, perform complicated and often nuanced tasks like operating aircrafts and maneuvering surgical instruments, all while effectively dismissing potential distractors like outputs from task-irrelevant instruments. There is a tremendous need to better understand and facilitate human cognitive performance under complex task conditions [4,5,6], particularly in specialized fields like aviation where pilot performance errors can have devastating consequences in both human life and economic ramifications. Indeed, recent statistics show that 80% of airplane accidents have been caused by human error, resulting in 523 deaths and $1.3B in damages in one year alone [7].

To understand the impact of complex task performance on human cognitive processing, it is essential to develop tools that enable the measurement of brain function under complex multi-dimensional task conditions that resemble complex naturalistic environments. Within the context of aviation, one commonly used technique is questionnaire-based behavioral assessments such as the NASA-TLX and Cooper–Harper Handling Quality Rating Scale [8,9,10]. These questionnaires have been employed for subjective assessments of cognition, especially cognitive workload, both as standalone measurements [11] and in conjunction with other metrics [12,13], in both aviation and other settings such healthcare [14,15]; however, they suffer from significant shortcomings. In particular, these tests are subjective, interfere with the primary task (e.g. flight task) at hand, and do not provide continuous time-resolved measurements as they can typically only be administered at the beginning or end of the task [16].

In order to address the concerns about the subjectivity of questionnaire-based assessments, several groups have employed a variety of physiological measurements in order to provide more objective assessments [17,18]. For example heart rate and heart rate variability measures have been employed for assessments of cognitive load in a variety of areas including aviation [19], clinical [20], and educational [21] settings. Previous works have also demonstrated the possibility of the non-contact measurement of heart rate using video analysis for the assessment of cognitive stress [22]. Yet others have explored the use of respiratory rate changes [23], pupil dilation [24], blink rates [25], and electrodermal measurements [26] for evaluation of cognition. Several prior works have also explored the possibility of utilizing one or more of the above-mentioned metrics as features for developing machine learning models as passive brain computer interfaces for classifying cognitive states (such as cognitive load, cognitive stress, level of trust or interest, etc.) [27,28].

While the physiological measurements described above provide objective measurements and are able to provide time-resolved assessments, they unfortunately do not provide direct measurements of the brain itself. On the other hand, neurophysiological assessments such as brainwave recordings using electroencephalography (EEG) have yielded metrics like quantitative EEG that examine the spectral content of brain activity within defined time windows, but these techniques do not provide information about the rapid temporal dynamics of neural activity, which occur on a millisecond scale and are essential to informing the brain’s response to various stimuli and task conditions [29,30,31]. Another EEG-based technique uses event-related potentials (ERPs) to capture specific brain responses elicited through externally driven sensory stimuli and can be used to assess brain temporal dynamics [32,33,34,35]. However, ERPs require a secondary task (e.g., sensory stimulation) to be superimposed on the already complex primary task (e.g., flight maneuvers), which interferes with the primary task of interest and creates artificial testing conditions that compromise the fidelity of the assessments. While some groups have employed machine learning classifiers with EEG-derived data for assessments of cognition in aviation, driving, human-machine interaction, and other domains [36,37,38,39], the primary challenge remains with the previously mentioned inability to seamlessly capture the underlying measures of brain dynamics within complex operational settings [40]. A better approach is needed that **provides objective measures of brain temporal dynamics during complex task performance** and can also **be seamlessly integrated into the underlying primary task**. Moreover, as complex task performances such as flight maneuvers require the integration of a broad spectrum of cognitive processes spanning the sensory, attention, memory, and information processing domains, the ideal brain function measure must also **enable the capture and evaluation of a wide range of neurocognitive responses**—all without interfering with underlying task performance.

Blink-related oscillations (BROs) are a recently discovered EEG-based brainwave response associated with spontaneous blinking (distinct from the well-known oculomotor effects). BROs represent environmental monitoring and awareness processes as the brain evaluates new visual information that appears following eye re-opening [41,42]. Although the natural phenomenon of spontaneous blinking has traditionally not been associated with cognitive processing, increasing evidence from behavioral studies have pointed to a potential link between blinking and cognition (e.g., blinking tends to occur at the ends of sentences when reading [43], speaker pauses when listening to speech [44], and scene changes when watching videos [45]). In support of such evidence, the neurophysiological BRO responses have been shown to index a broad spectrum of neurocognitive processes spanning the sensory, information processing, and episodic memory domains and also engage key regions in the brain associated with environmental monitoring and awareness, visuospatial processing, and episodic memory [41,46,47,48]. BRO responses are observable under resting conditions, and they are also modulated by task and environmental factors. Indeed, studies have shown that spontaneous blinking during a cognitive loading task (e.g., mental arithmetic without sensory stimulation) results in decreased cortical activations compared to blinking during rest, suggesting reduced availability of neuronal resources for blink-related processing under cognitive loading conditions [46]. Additionally, spontaneous blinking under external sensory stimulation (e.g., ongoing dynamic auditory vs. visual inputs) produces altered temporal and spectral BRO response features that are consistent with the brain’s dynamic adaption of blink processing for accommodating differential sensory requirements [47]. Crucially, the BRO response modulations are independent of behavioral characteristics such as blink rate, indicating that BRO effects represent neurocognitive mechanisms underlying blink-related information processing that are distinct from peripheral kinematics. Together, these findings create an intriguing possibility that the simple act of spontaneous blinking may represent a previously unexplored window into brain function—and that BRO responses may hold the key to understanding human cognitive performance in complex task conditions like flight maneuvers, with the unique advantage of enabling natural and seamless integration into the existing environment.

Previous studies have explored the behavior of BRO responses under conditions that varied one factor at a time, i.e., changing cognitive load or changing sensory environment. For BROs to be applicable to naturalistic assessments, additional evaluations under situations that more closely resemble the complex real-world environments need to be undertaken. That is, BRO responses need to be evaluated under conditions in which simultaneous sensory, cognitive, and motor demands are present. One preliminary study evaluated BRO responses using the N-back task which combines sensory, cognitive loading and motor response requirements, and reported the presence of BRO responses and their ability to capture cognitive loading differences [49]. While these results are promising, for BRO responses to fulfill their potential of providing assessments in naturalistic settings, they have to be evaluated not only in sanitized classic laboratory tasks such as N-back, but under realistic multi-tasking conditions as well. But no study has evaluated the BRO response within multi-tasking situations.

The aviation domain presents a prime example of such task conditions, with a multitude of simultaneous sensory (e.g., visual, auditory, and tactile inputs), cognitive (e.g., visuospatial processing, memory, arithmetic, and language), as well as motor (e.g., throttle control) requirements for pilot performance in the cockpit [50]. Prior to evaluations within actual aircraft environments, for participant safety and improved scientific validity (e.g., repeatability of settings across participants), it is beneficial to evaluate BRO responses using well-established models or simulations. The Multi-Attribute Task Battery (MATB), simulates benchmarked complex and realistic tasks analogous to those undertaken by pilots/aircrews in flight [51]. MATB is well-established as a computer-based task battery that effectively simulates multi-tasking through its constituent tasks consisting of system monitoring, tracking, communications, resource management, and scheduling [52]. While several studies have utilized traditional EEG-based measurements (e.g., power in canonical frequency bands ) to assess cognitive constructs such as workload and situational awareness using MATB tasks [53,54,55], no study has evaluated the potential of BRO responses to assess cognition while participants complete MATB tasks. This study evaluated BRO responses while adult human participants completed the MATB under various task difficulty levels. We hypothesized that (1) BRO responses would be present during the complex multi-tasking conditions simulated by the MATB, and (2) BRO responses would be modulated by task difficulty.

## 2. Methods

### 2.1. Dataset Details

The data utilized in the current study were collected by the US Air Force Research Laboratory and provided for the Cognitive State Assessment Competition 2011. A total of 19 channels of EEG data were collected. Additionally, vertical and horizontal electrooculograms (VEOG and HEOG) were collected using bipolar electrodes, located above and below the left eye and the outer canthus of each eye respectively. All data were collected using the MICROAMPS system from SAM Technologies, Inc. (San Francisco, CA, USA), with default high-pass and low-pass filters at 0.05 Hz and 100 Hz, respectively, and a sampling rate of 256 Hz. All electrodes were made of tin, placed according to the international 10/20 system, and skin–electrode impedances were maintained below 5 kΩ for EEG and 15 kΩ for EOG channels. All EEG channels were referenced to a single left mastoid electrode. Additional details are provided elsewhere [56].

### 2.2. Participant and Task Details

**Figure 1:**
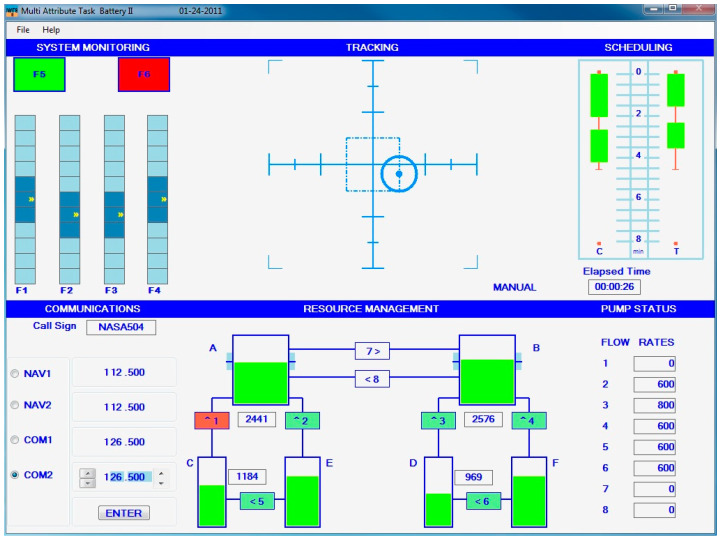
An overview of the Multi-Attribute Task Battery II task. Image from National Aeronautics and Space Administration (NASA): https://matb.larc.nasa.gov (accessed on 30 October 2023).

Data from 8 healthy adult participants (mean age = 21.1 years, 5 females) were collected over 5 separate days with each participant completing 3 repetitions per day. Data were collected while participants completed the MATB task, which involves the participant completing four tasks simultaneously, representative of activities that pilots may perform during flight. These include a tracking task, monitoring pressure gauges and warning lights, air traffic control communications, and resource allocation tasks (see Figure 1). In each repetition, segments of task difficulty intended to produce low, medium, and high workload (for example, by varying the rate of change of the target to be tracked in the tracking task) were presented in a random order, with transition time between workload segments. The five days of data collection for each participant were not sequential but spread out over the course of one month. The reader is directed to prior descriptions of the dataset for additional details [56,57].

### 2.3. Data Processing

The data were analyzed using a combination of EEGLAB [58] and custom processing software using MATLAB 2020b (Mathworks Inc., Natick, MA, USA). Data were band-pass filtered (0.1–80 Hz) using a zero-phase 4th-order Butterworth filter, notch filtered (60 Hz) to remove line noise, and visually inspected for removal of artifactual segments. All data from the 3 repetitions per day and 5 days of collection were utilized in the analyses, separated by workload levels.

#### 2.3.1. Blink Identification and Blink Rate

Data from the VEOG channel were low-pass filtered (30 Hz), and a convolution-based, semi-automated template matching procedure as described in prior BRO works [47,48] was utilized to identify blink instances. For calculation of blink rates, the identified blink instances were grouped into low, medium and high workload conditions and statistical comparisons across workload levels were undertaken using one-way repeated measures analysis of variance (ANOVA) with Huynh–Feldt correction for violations of sphericity. Post hoc analyses were conducted with *t*-test with Bonferroni correction for multiple comparisons.

#### 2.3.2. EEG Denoising and Effectiveness of Artifact Removal

Independent component analysis (ICA) was performed using the InfoMax algorithm in accordance with prior published procedures [41,59,60,61,62], and components corresponding to artifact (e.g., blinks, saccades, muscle contractions, cardiac activity, and breathing) were removed. To ensure complete artifact removal prior to extracting BROs, the effectiveness of the ICA-based denoising procedure was critically evaluated using both qualitative and quantitative approaches. Qualitative assessments were performed by examining individual-level, trial-averaged blink spatial topography maps to verify the elimination of known temporal and topographical features of the ocular artifact and to ensure residual signals were due to BRO activity. Quantitative assessments utilized the ocular contamination index (OCI) [47,63], which measures the signal amplitude at the time of blink maximum (denoted 0 ms in figures) relative to baseline levels (denoted −1000 ms figures) pre- and post-denoising. Pre- and post-denoising OCI values were statistically compared using two-tailed *t*-test.

### 2.4. Time Domain Analysis

Denoised continuous data were segmented into 3 s epochs centered on each blink instance that passed temporal thresholding (>3 s separation from adjoining blink instances) in line with prior BRO literature [41,46,47,48]. Data were then filtered into delta (0.5–4 Hz) band to derive time-domain BRO responses. To statistically validate that the BRO phenomenon exists, the mean amplitude at the Pz electrode during baseline interval (−1300 ms to −1100 ms pre-blink), mean amplitude during the interval overlapping the first peak of the BRO response (200 ms to 400 ms post-blink), and mean amplitude during interval overlapping the second identified peak (450 ms to 650 ms post-blink) were computed and compared for each workload level using repeated-measures analysis of variance (ANOVA), followed by post hoc *t*-tests with Bonferroni correction.

Additionally, for assessing the ability of BRO responses to capture cognitive workload/task difficulty differences, the analyses focused on the P3, Pz, P4, O1, and O2 electrode signals due to their proximity to the precuneus region, which is known to be a key source of the BRO response. Specifically, mean amplitude in 100 ms time windows surrounding the visually identified peaks of interest in the BRO responses (at −500 ms, 150 ms, 300 ms, and 500 ms) were calculated and compared using repeated-measures ANOVA with factors of *channel* and *workload* for each peak time, and number of blink instances used as a covariate to account for any potential signal-to-noise differences. Post hoc comparisons using *t*-tests were conducted with Bonferroni correction to account for multiple comparisons.

### 2.5. Frequency Domain Analysis

Time–frequency analysis was used to explore blink-related changes in the spectral content of BRO responses. In particular, in line with prior works, time–frequency analysis was performed using continuous wavelet transform (CWT) with the Morlet function with six cycles. CWT was carried out for each blink instance at the Pz electrode, and the log power was obtained as the logarithm of the squared absolute values of the coefficients for each frequency. Baseline correction was performed by subtracting from each trial the mean log power within the baseline interval of −1500 ms to −500 ms relative to blink instance. The baseline corrected CWT coefficients were grouped by workload level, and in line with prior works [47,48], permutation-based statistics were utilized to both assess the presence of expected BRO features in the time–frequency plane as well as to compare across workload levels. In particular, for assessment of BRO feature presence, the pre-blink and post-blink sections of the time–frequency plane were compared using a *t*-statistic approach, and then the groupings of pre-blink and post-blink segments were randomly permuted 1000 times to generate a null distribution of *t*-statistic values against which the true *t*-statistic value was compared for significance. A similar approach of permutated groupings was utilized to assess for significant differences across workload levels with Bonferroni correction to account for multiple comparisons.

## 3. Results

### 3.1. Blink Behaviour

Blink rate decreased as workload increased (F(1.61,192.12) = 102.97, *p* < 0.001). The average blink rates across all sessions were 19.4 ± 11.2, 16.0 ± 7.6, and 11.9 ± 6.4 blinks per minute for low, medium, and high workloads, respectively. Significant differences in blink rates were observed between low and high (t(119) = 11.98, *p* < 0.001) and medium and high (t(119) = 10.48, *p* < 0.001) workload levels, as well as low and medium workload levels (t(119) = 6.46, *p* < 0.001).

### 3.2. Artifact Removal

**Figure 2:**
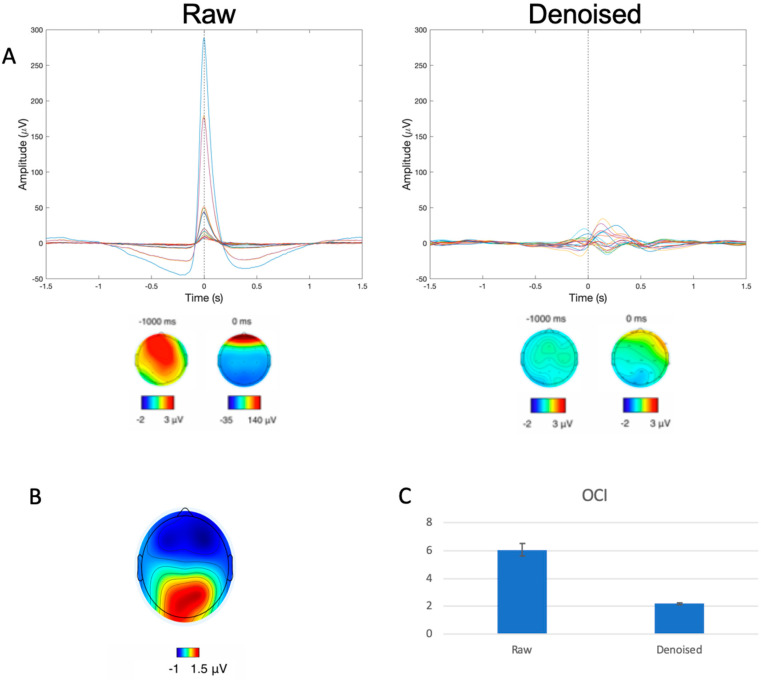
(**A**) Representative participant data before and after artifact removal of all trial-averaged EEG channels with corresponding spatial topographies. Dotted line corresponds to time of blink event, and colored lines represent the various channels of data. Spatial topography plots underneath demonstrate the distribution of signal power at −1000 ms and 0 ms, showcasing the large frontally focused signal power distribution corresponding to blinking in the raw (left) but not the cleaned (right) data. (**B**) Spatial topography at 340 ms post-blink demonstrating the concentration of signal power at the posterior locations consistent with BRO response patterns. (**C**) OCI values from raw and denoised (cleaned) data, shown as mean ± SEM.

As shown in Figure 2A (left), prior to denoising, the raw data exhibited patterns consistent with the presence of large ocular artifacts in the form of a large peak (>250 μV) in the time domain signal and frontally concentrated signal power in the scalp topography plot at the time of the blink maximum (denoted 0 ms in the plot). Subsequent to denoising, these features were no longer present in the cleaned data as evidenced by Figure 2A (right) by way of the removal of the large signal spike and the lack of frontally concentrated power in the scalp topography at 0 ms. In addition to the visually apparent qualitative changes, quantitative assessments using OCI also demonstrated the efficacy of the ICA-based denoising, with significant reduction in OCI values from pre- (6.07 ± 4.87) to post-denoising (2.18 ± 0.86) (t(117) = 8.519, *p* < 0.001).

### 3.3. Time Domain Analysis

**Figure 3:**
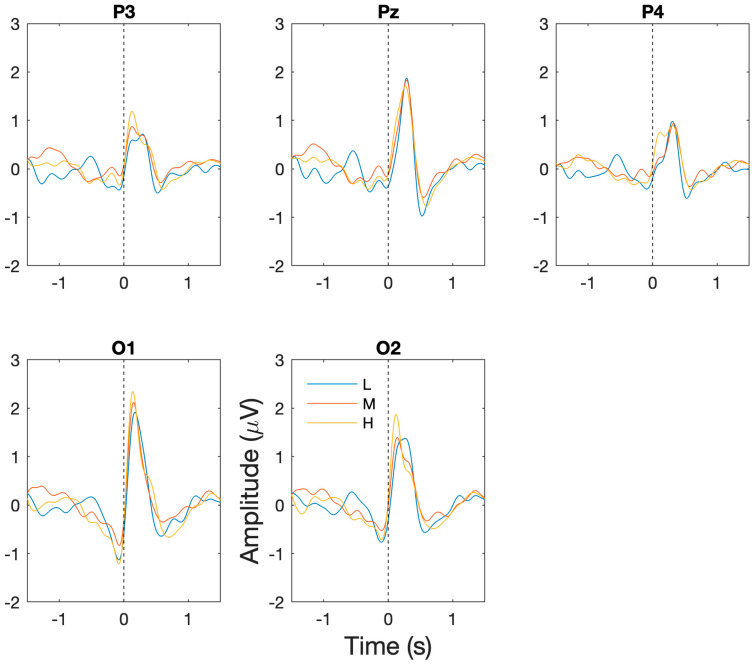
Grand averaged BRO response waveforms across all participants and sessions at P3, PZ, P4, O1, and O2 electrodes. Dotted vertical line corresponds to time when blink occurs. *Blue = low workload (L), red = medium workload (M), yellow = high workload (H)*.

The successful capture of BROs: for each of the workload/task difficulty levels, the presence of the expected BRO responses were confirmed through significant increases in post-blink BRO response magnitude relative to the corresponding baseline periods. Specifically, as shown in Figure 3, a dual-peak morphology was observed corresponding to the first peak C1 and the second peak C2 in comparison to the baseline period (−1300 to −1100 ms) for low (F(2,14) = 50.9, *p* < 0.001), medium (F(2,14) = 36.7, *p* < 0.001), and high (F(2,14) = 29.3, *p* < 0.001) workload levels at the Pz electrode. The presence of the BRO response features were confirmed through post hoc analyses demonstrating significantly more positive signal amplitude for C1 (low: t(7) = 9.2, *p* < 0.001, med: t(7) = 4.3, *p* = 0.004, high: t(7) = 5.9, *p* = 0.001) and a more negative amplitude for C2 (low: t(7) = 2.93, *p* = 0.02, med: t(7) = 6.7, *p* < 0.001, high: t(7) = 3.3, *p* = 0.01) relative to baseline.

Workload/task difficulty differences: additionally, in order to assess the ability of BRO responses to capture changes in task difficulty, comparisons of the BRO responses at the P3, Pz, P4, O1, and O2 electrodes were undertaken across workload levels. For the pre-blink peak at −500 ms, there was no significant main effect of channel or an interaction effect, but a significant main effect of workload was present (F(2,14) = 5.53, *p* = 0.02). Post hoc analyses showed significantly reduced signal amplitude for high compared to low workload at the O2 electrode (*p* = 0.009) and reduction in medium from low workload conditions at the O1 electrode (*p* = 0.037). For the 150 ms peak, significant main effects of channel (F(3.26,22.87) = 4.69, *p* = 0.009) and workload (F(2,14) = 4.42, *p* = 0.02) were present but no significant interaction effect was identified. Post hoc analyses revealed significantly lower amplitude at the P4 electrode compared to the P3 electrode (*p* = 0.03) and a significant increase in BRO signal amplitude for high workload relative to low workload conditions at the P3 (*p* = 0.004), Pz (*p* = 0.04) and P4 (*p* = 0.023) electrodes. For the 300 ms peak, a significant main effect of channel (F(4,28) = 9.73, *p* < 0.001) and an interaction effect of channel and workload (F(8,56) = 3.71, *p* = 0.02) was present. Post hoc analyses captured significant differences in overall amplitudes between P3 and Pz (*p* < 0.001) and Pz and P4 (*p* = 0.003); however, no significant differences were present for workload levels at any electrode location. For the 500 ms post-blink peak, significant main effects of channel (F(2,14) = 2.75, *p* = 0.48) and workload (F(1.18,8.25) = 6.71, *p* = 0.009) were present, but no significant interaction effect was present. Post hoc analyses revealed significantly more negative amplitude at the O1 electrode for the medium workload compared to the low workload (*p* = 0.035).

### 3.4. Frequency Domain Analysis

**Figure 4:**
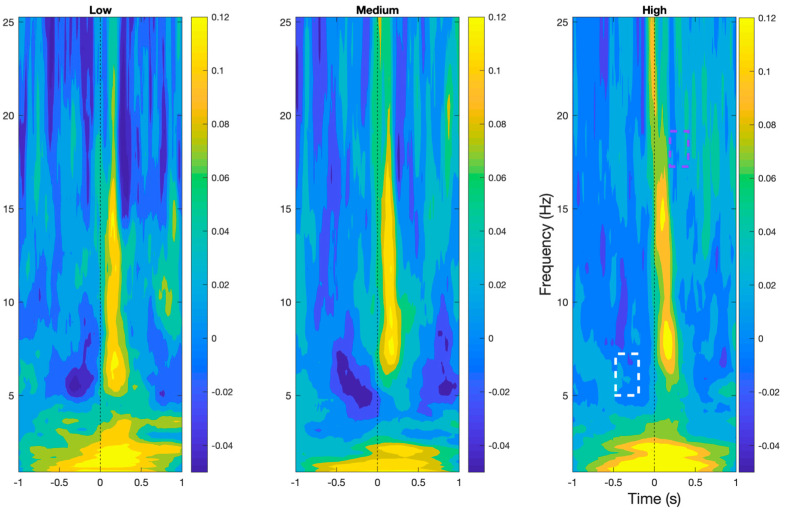
BRO responses in time–frequency plane at the Pz electrode demonstrating the characteristic features of the BRO phenomenon across all workload levels. Purple box denotes region of significant reduction in beta desynchronization in high compared to low workload. White box denotes regions of significant reduction in theta desynchronization in high workload compared to the other levels.

As shown in Figure 4, Time–frequency analyses demonstrated the presence of prototypical BRO features, namely early delta (0.5–4 Hz) and beta (15–30 Hz) band event-related synchronization, followed by theta (4–7 Hz) and alpha band (8–12 Hz) event-related desynchronization in all workload levels within the post-blink period (*p* < 0.05). In addition, theta band desynchronization was also observed around 500 ms pre-blink.

Comparisons across workload levels demonstrated a significant reduction in beta band desynchronization approximately 300 ms post-blink and a significant reduction in desynchronization of the theta band approximately 500 ms pre-blink in the high workload condition compared to the low and medium workload levels (*p* < 0.05).

## 4. Discussion

### 4.1. Main Findings

In this study, we examined blink-related oscillation responses while participants completed multiple complex tasks that engaged several cognitive domains simultaneously. As expected, blink behavior measures indicated a decrease in blink rate with increasing task difficulty/cognitive load. In support of our hypotheses, we observed that BRO responses were indeed present in both time and frequency domains within such a complex multitasking situation (Hyp. 1), and these responses reflected changes in cognitive loading or task difficulty in both the time and frequency domains (Hyp. 2). Additionally, differences in pre-blink cognitive activity, specifically in the form of decreasing theta band desynchronization, were also observed in the high workload condition.

### 4.2. Presence of BRO Phenomenon

As the first study examining the BRO response within a complex multitasking situation, our results in the current study indicate that the expected pattern of BRO features reported in prior works [47,48] are indeed present. In particular, within the time domain, a two-peak complex in the post-blink period as reported in previous literature was also observed in the current study. However, it is important to note that while the two-peak complex was observed, the polarities of the components were different from previous reports, but this could potentially be due to the differing reference locations/montages, as the previous reports utilized an average reference [47], whereas the current study utilized a left mastoid reference. In line with this reasoning, prior studies have demonstrated that differing reference locations can impact the observed neural activity on scalp EEG [64,65].

Time–frequency analysis revealed the presence of a cascade of beta and delta band synchronization, followed by theta and alpha band desynchronization post-blink, consistent with previous reports of the BRO phenomenon [41,46,47,48,66,67]. Indeed, previous literature has postulated the role of early beta synchronization in the sensory processing of the visual scene presented subsequent to the eyes re-opening, as well as delta synchronization in long-range cortical communication, followed by alpha and theta activity representing higher order cognitive functions by way of episodic memory and information processing [46,47]. Importantly, in the current study, the same patterns of activity were present in time and frequency domains across all task difficulty levels of the MATB, further confirming the presence of the BRO effect within the multitasking environment.

### 4.3. Capturing Cognitive Load Changes

Blink rate decreased with increasing cognitive load, confirming the successful elicitation of differential cognitive loads by the MATB. These results are in line with previous work that utilized the same dataset and reported a similar pattern of decrease in blink rate [68]. Similar decreases in blink rates have previously been reported across a variety of tasks that modulate cognitive load [69,70,71], especially when using digital screens [72]. In addition, changes in blink rate have also been associated with cognition in other domains such as human infant development [73], individual-level engagement in scene content [74], and even clinical diagnosis [75].

In the time domain, increasing cognitive load resulted in decreased BRO responses in the pre-blink (~−500 ms) and post-blink (~500 ms) intervals at the occipital electrodes. These findings are in agreement with prior literature using BRO responses which have also demonstrated a decrease in signal amplitude with increased cognitive loading [46,49]. Given that these effects were observed at the occipital electrodes, it may reflect the competing visual processing demands when someone is engaged in the (visually) challenging MATB task and the brain has to process additional blink-related visual information, resulting in the reduced availability of neural resources for blink information processing.

Additionally, increases in cognitive load also resulted in an increase in BRO amplitude at the parietal electrodes at approximately 150 ms post-blink. Since BROs are considered to reflect the brain’s processing of the visual scene after the eyes reopen, this increase in BRO response potentially reflects increased visuospatial processing that is required to process the new visual scene that appears after the eyes re-open. Given that the higher load condition has more complex visual information compared to low load, this would entail more visuospatial processing, especially by areas such as the precuneus (located adjacent to the Pz electrode) which is heavily implicated in blink-related environmental and visuospatial processing functions [76,77]. In line with this reasoning, these increases in BRO responses are also in agreement with findings from a recent study that demonstrated the enhancement of brain responses at posterior electrodes when people were presented with higher numbers of landmarks during a map-assisted navigation task (i.e., increased visuospatial information processing demands) [78].

In the frequency domain, changes in cognitive load manifested as a post-blink decrease in beta desynchronization (~300 ms) and a pre-blink decrease in theta desynchronization (~−400 ms). Beta band power suppression (desynchronization) has been linked with increased levels of cortical processing in a variety of experimental paradigms including motor movement [79], language processing [80,81], information retrieval [82], and working memory [83]. In the current study, we speculate that the beta desynchronization is similarly reflective of the cortical processing of blink-related information, and the decrease in beta desynchronization potentially reflects the reduced availability of neuronal resources for blink-related information processing under the high workload condition when the brain is already busy with task-related information processing corresponding to the tasks in the MATB.

Theta band desynchronization has previously been associated with increasing task difficulty in spatial working memory tasks, especially during the retention phase [84]. Much like theta band desynchronization within spatial working memory tasks tracks the requirement of participants to retain the location of various stimuli, we speculate that the pre-blink theta desynchronization in the current study reflects the brain’s additional efforts in studying the ‘scene’ ahead of an anticipated upcoming blink event when momentary loss of visual input will occur. Such anticipatory neurocognitive mechanisms further strengthen the evidence for the role of BROs in environmental processing [41,66], with the brain dynamically balancing the need for capturing/remembering sensory information (i.e., visual stimuli from MATB tasks in this case) with the visual input loss caused by a blink. Such behavior has previously been hypothesized to provide an evolutionary advantage for anti-predator surveillance [47,85]. In parallel with the observed decrease in beta band desynchronization observed in this study, the decrease in pre-blink theta desynchronization could also be reflective of the reduced availability of neuronal resources under the high workload condition when the brain is already busy processing information related to the very demanding MATB tasks.

### 4.4. Caveats and Future Directions

While the results of the current study are promising, the study does have some limitations. First, the results are based on data from eight participants, and so future studies are needed to further validate these findings in a larger sample size. The small sample size also potentially introduces concerns about the lack of diversity among the participant pool, the impact on participant behavior due to use of the equipment, and the applicability to a larger population, further amplifying the need for replication in a larger sample size. Second, while the participants practiced until they reached a plateau for performance in MATB prior to the collection of EEG data, it is impossible to rule out if any additional learning occurred over the period of five data collection days, which may impact the BRO responses reported here. Furthermore, while the current study utilized data from a whole-head 19-channel EEG system, future works should evaluate the findings using lower density and alternate location sensors [86,87,88] that enable easier translation into real-world naturalistic settings (e.g., actual aircraft cockpits [89]) when paired with appropriate signal processing techniques [48,90,91,92]. 

## 5. Conclusions

This study demonstrated the successful capture of BRO responses within a very complex multitasking situation that closely resembles the modern increasingly complex environment that we operate in. In addition to the evidence of their presence, BRO responses were also shown to reflect the level of task difficulty, potentially creating an avenue for cognitive state assessments using BRO responses within complex environments—ranging from highly specialized ones such as aircraft cockpits to everyday settings such as driving—without the need for artificial test setups, thereby enabling seamless integration into the environment of interest. Furthermore, the results also show that BRO responses are sensitive to the brain’s dynamic reallocation of neurocognitive mechanisms, thereby opening a new frontier in our ability to better understand brain function within naturalistic settings without the need for sanitary laboratory tasks that limit our ability to assess complex cognitive phenomenon.

## Data Availability

Data that underlie the findings of this study were made available for the CSAC 2011 competition and can be requested by contacting the US Air Force Research Lab (Justin Estepp, justin.estepp@wpafb.af.mil).
